# Supplementary material for: Perinatal and maternal factors associated with Autism Spectrum Disorder
Source: PLoS One. 2026 Mar 18;21(3):e0316968. doi: 10.1371/journal.pone.0316968 (PMC12998875; doi:10.1371/journal.pone.0316968)
Supplement: S1 Table — (DOCX) [file pone.0316968.s001.docx]

**Table s1 (supporting material). Sex by ASD severity, intellectual disability, and familial history for ASD.**

|  |  | Males N=819 | | Females N=177 | |
| --- | --- | --- | --- | --- | --- |
|  |  | n | (%) | n | (%) |
| Severity of ASD | |  |  |  |  |
|  | Mild | 628 | (76.7) | 126 | (71.2) |
|  | Moderate | 146 | (17.8) | 34 | (19.2) |
|  | Severe | 45 | ( 5.5) | 17 | ( 9.6) |
| Intellectual disability | |  |  |  |  |
|  | No | 612 | (83.0) | 125 | (17.0) |
|  | Yes (confirmed or suspected) | 207 | (79.9) | 52 | (20.1) |
| Familial history | |  |  |  |  |
|  | Non familial ASD | 595 | (82.2) | 129 | (17.8) |
|  | Familial ASD | 224 | (82.4) | 48 | (17.6) |
