# Supplementary material for: Perinatal and maternal factors associated with Autism Spectrum Disorder
Source: PLoS One. 2026 Mar 18;21(3):e0316968. doi: 10.1371/journal.pone.0316968 (PMC12998875; doi:10.1371/journal.pone.0316968)
Supplement: S2 Table — (DOCX) [file pone.0316968.s002.docx]

**Table s2. Familial and non-familial ASD in relation to presence of intellectual disability among children with ASD.**

|  | No intellectual disability suspected | | Confirmed/suspected intellectual disability | | p-value |
| --- | --- | --- | --- | --- | --- |
|  | n | ( % ) | n | ( % ) |  |
| Non-familial ASD | 527 | (72.8) | 197 | (27.2) | 0.157 |
| Familial ASD | 210 | (77.2) | 62 | (22.8) |  |
| Total | 737 | (74.0) | 259 | (26.0) |  |
