# Supplementary material for: Perinatal and maternal factors associated with Autism Spectrum Disorder
Source: PLoS One. 2026 Mar 18;21(3):e0316968. doi: 10.1371/journal.pone.0316968 (PMC12998875; doi:10.1371/journal.pone.0316968)
Supplement: S3 Table — (DOCX) [file pone.0316968.s003.docx]

**Table S3. Children with ASD and controls by matching criteria (year of birth and sex), and maternal characteristics.**

|  |  | **Children with ASD N=996** | | **Controls N=9960** | |  | **Odds Ratio** | | **Adjusted OR*** | |
| --- | --- | --- | --- | --- | --- | --- | --- | --- | --- | --- |
|  |  | **n** | **(%)** | **n** | **(%)** |  | **OR** | **95% CI** | **OR** | **95% CI** |
| **Maternal age** | |  |  |  |  |  |  |  |  |  |
|  | <20 | 21 | ( 2.1) | 170 | ( 1.7) |  | 1.24 | 0.77-2.01 | 0.98 | 0.61-1.57 |
|  | 20-34 | 775 | (77.8) | 7800 | (78.3) |  | 1.00 | Reference | 1.0 | Reference |
|  | 35-39 | 156 | (15.7) | 1682 | (16.9) |  | 0.91 | 0.75-1.10 | 0.97 | 0.80-1.16 |
|  | 40+ | 44 | ( 4.4) | 308 | ( 3.1) |  | 1.36 | 0.96-1.93 | **1.46** | **1.05-2.02** |
| **Parity** | |  |  |  |  |  |  |  |  |  |
|  | 1 | 518 | (52.0) | 4765 | (47.8) |  | **1.21** | **1.06-1.39** | **1.23** | **1.07-1.41** |
|  | 2+ | 478 | (48.0) | 5195 | (52.2) |  | 1.0 | Reference | 1.0 | Reference |
| **Maternal smoking** | |  |  |  |  |  |  |  |  |  |
|  | Yes | 138 | (13.9) | 940 | ( 9.4) |  | **1.55** | **1.27-1.89** | **1.49** | **1.22-1.82** |
|  | No | 826 | (82.9) | 8748 | (87.8) |  | 1.00 | Reference | 1.0 | Reference |
|  | *Not known* | 32 | ( 3.2) | 272 | ( 2.7) |  | 0.86 | 0.46-1.60 | 1.01 | 0.68-1.50 |
| **Maternal BMI** | |  |  |  |  |  |  |  |  |  |
|  | <18.5 | 21 | ( 2.1) | 184 | ( 1.8) |  | 1.55 | 0.98-2.46 | 1.53 | 0.96-2.43 |
|  | 18.5-24.9 | 434 | (43.6) | 5404 | (54.3) |  | 1.0 | Reference | 1.0 | Reference |
|  | 25-29.9 | 280 | (28.1) | 2524 | (25.3) |  | **1.40** | **1.20-1.64** | **1.41** | **1.20-1.65** |
|  | 30+ | 178 | (17.9) | 1269 | (12.7) |  | **1.79** | **1.49-2.16** | **1.76** | **1.46-2.12** |
|  | *Not known* | 83 | ( 8.3) | 579 | ( 5.8) |  | **1.78** | **1.39-2.28** | **1.78** | **1.36-2.31** |
| **Involuntary childlessness** | | |  |  |  |  |  |  |  |  |
|  | No (<2 years) | 938 | (94.2) | 9438 | (94.8) |  | 1.00 | Reference | 1.0 | Reference |
|  | 2-4 years | 44 | ( 4.4) | 416 | ( 4.2) |  | 1.08 | 0.78-1.50 | 1.02 | 0.71-1.45 |
|  | 5 years or more | 14 | ( 1.4) | 106 | ( 1.1) |  | 1.14 | 0.61-2.12 | 1.14 | 0.61-2.10 |
| **Assisted reproduction** | | |  |  |  |  |  |  |  |  |
|  | Yes | 30 | ( 3.0) | 278 | ( 2.8) |  | 1.08 | 0.74-1.58 | 1.02 | 0.64-1.60 |
|  | No | 966 | (97.0) | 9682 | (97.2) |  | 1.0 | Reference | 1.0 | Reference |
