# Supplementary material for: Perinatal and maternal factors associated with Autism Spectrum Disorder
Source: PLoS One. 2026 Mar 18;21(3):e0316968. doi: 10.1371/journal.pone.0316968 (PMC12998875; doi:10.1371/journal.pone.0316968)
Supplement: S4 Table — (DOCX) [file pone.0316968.s004.docx]

**Table s4. Autism severity, presence of intellectual disability, and familial history, respectively, by maternal characteristics**

|  |  | Mild ASD  N=754 | | Moderate/ severe ASD  N=242 | | ASD without ID N=737 | | ASD with ID N=259 | | Non-familial ASD  N=724 | | Familial ASD N=150 | | Controls N=9960 | |
| --- | --- | --- | --- | --- | --- | --- | --- | --- | --- | --- | --- | --- | --- | --- | --- |
|  |  | n | ( % ) | n | ( % ) | n | ( % ) | n | ( % ) | n | ( % ) | n | ( % ) | n | ( % ) |
| Maternal age | |  |  |  |  |  |  |  |  |  |  |  |  |  |  |
|  | <20 | 16 | ( 2.1) | 5 | ( 2.1) | 13 | ( 1.8) | 8 | ( 3.1) | 13 | ( 1.8) | 2 | ( 1.3) | 170 | ( 1.7) |
|  | 20-34 | 609 | (80.8) | 166 | (68.6) | 584 | (79.2) | 191 | (73.7) | 569 | (78.6) | 110 | (73.3) | 7800 | (78.3) |
|  | 35-39 | 104 | (13.8) | 52 | (21.5) | 109 | (14.8) | 47 | (18.1) | 117 | (16.2) | 30 | (20.0) | 1682 | (16.9) |
|  | 40+ | 25 | ( 3.3) | 19 | ( 7.9) | 31 | ( 4.2) | 13 | ( 5.0) | 25 | ( 3.5) | 8 | ( 5.3) | 308 | ( 3.1) |
| Parity |  |  |  |  |  |  |  |  |  |  |  |  |  |  |  |
|  | Primiparity | 404 | (53.6) | 114 | (47.1) | 397 | (53.9) | 121 | (46.7) | 416 | (57.5) | 31 | (20.7) | 4765 | (47.8) |
|  | Multiparity | 350 | (46.4) | 128 | (52.9) | 340 | (46.1) | 138 | (53.3) | 308 | (42.5) | 119 | (79.3) | 5195 | (52.2) |
| Maternal smoking | |  |  |  |  |  |  |  |  |  |  |  |  |  |  |
|  | Yes | 110 | (14.6) | 28 | (11.6) | 108 | (14.7) | 30 | (11.6) | 97 | (13.4) | 21 | (14.0) | 940 | ( 9.4) |
|  | No | 618 | (82.0) | 208 | (86.0) | 604 | (82.0) | 222 | (85.7) | 600 | (82.9) | 127 | (84.7) | 8748 | (87.8) |
|  | *Not known* | 26 | ( 3.4) | 6 | ( 2.5) | 25 | ( 3.4) | 7 | ( 2.7) | 27 | ( 3.7) | 2 | ( 1.3) | 272 | ( 2.7) |
| Maternal BMI | |  |  |  |  |  |  |  |  |  |  |  |  |  |  |
|  | <18.5 | 16 | ( 2.1) | 5 | ( 2.1) | 12 | ( 1.6) | 9 | ( 3.5) | 14 | ( 1.9) | 5 | ( 3.3) | 184 | ( 1.8) |
|  | 18.5-24.9 | 323 | (42.8) | 111 | (45.9) | 335 | (45.5) | 99 | (38.2) | 318 | (43.9) | 62 | (41.3) | 5404 | (54.3) |
|  | 25-29.9 | 214 | (28.4) | 66 | (27.3) | 189 | (25.6) | 91 | (35.1) | 204 | (28.2) | 44 | (29.3) | 2524 | (25.3) |
|  | 30+ | 139 | (18.4) | 39 | (16.1) | 144 | (19.5) | 34 | (13.1) | 126 | (17.4) | 28 | (18.7) | 1269 | (12.7) |
|  | *Not known* | 62 | ( 8.2) | 21 | ( 8.7) | 57 | ( 7.7) | 26 | (10.0) | 62 | ( 8.6) | 11 | ( 7.3) | 579 | ( 5.8) |
| Involuntary childlessness | | |  |  |  |  |  |  |  |  |  |  |  |  |  |
|  | No (<2 years) | 711 | (94.3) | 227 | (93.8) | 688 | (93.4) | 250 | (96.5) | 677 | (93.5) | 145 | (96.7) | 9438 | (94.8) |
|  | 2-4 years | 33 | ( 4.4) | 11 | ( 4.5) | 39 | ( 5.3) | 5 | ( 1.9) | 35 | ( 4.8) | 3 | ( 2.0) | 416 | ( 4.2) |
|  | 5+years | 10 | ( 1.3) | 4 | ( 1.7) | 10 | ( 1.4) | 4 | ( 1.5) | 12 | ( 1.7) | 2 | ( 1.3) | 106 | ( 1.1) |
| Assisted reproduction | |  |  |  |  |  |  |  |  |  |  |  |  |  |  |
|  | Yes | 22 | ( 2.9) | 8 | ( 3.3) | 24 | ( 3.3) | 6 | ( 2.3) | 25 | ( 3.5) | 2 | ( 1.3) | 278 | ( 2.8) |
|  | No | 732 | (97.1) | 234 | (96.7) | 713 | (96.7) | 253 | (97.7) | 699 | (96.5) | 148 | (98.7) | 9682 | (97.2) |
