# Supplementary material for: Perinatal and maternal factors associated with Autism Spectrum Disorder
Source: PLoS One. 2026 Mar 18;21(3):e0316968. doi: 10.1371/journal.pone.0316968 (PMC12998875; doi:10.1371/journal.pone.0316968)
Supplement: S5 Table — (DOCX) [file pone.0316968.s005.docx]

**Table s5. Autism severity, presence of intellectual disability, and familial history, respectively, by maternal characteristics.**

|  |  | **Mild ASD vs controls** | | **Moderate/Severe ASD vs controls** | |  | **ASD without ID vs controls** | | **ASD with ID vs controls** | |  | **Non-familial^a^ ASD vs controls** | | **Familial^b^ ASD vs controls** | |
| --- | --- | --- | --- | --- | --- | --- | --- | --- | --- | --- | --- | --- | --- | --- | --- |
|  |  | **AOR*** | **95% CI** | **AOR*** | **95% CI** |  | **AOR*** | **95% CI** | **AOR*** | **95% CI** |  | **AOR*** | **95% CI** | **AOR*** | **95% CI** |
| **Maternal age** | |  |  |  |  |  |  |  |  |  |  |  |  |  |  |
|  | <20 | 0.91 | 0.54-1.55 | 1.24 | 0.49-3.12 |  | 0.77 | 0.43-1.38 | 1.72 | 0.82-3.66 |  | 0.77 | 0.43-1.37 | 1.83 | 0.86-3.87 |
|  | 20-34 | 1.00 | Reference | 1.00 | Reference |  | 1.00 | Reference | 1.00 | Reference |  | 1.00 | Reference | 1.00 | Reference |
|  | 35-39 | 0.83 | 0.66-1.03 | **1.47** | **1.06-2.03** |  | 0.91 | 0.73-1.28 | 1.14 | 0.82-1.61 |  | 1.04 | 0.84-1.28 | 0.81 | 0.57-1.15 |
|  | 40+ | 1.06 | 0.69-1.61 | **2.86** | **1.74-4.71** |  | 1.38 | 0.94-2.02 | 1.68 | 0.94-3.01 |  | 1.19 | 0.78-1.81 | **2.06** | **1.26-3.37** |
| **Parity** | |  |  |  |  |  |  |  |  |  |  |  |  |  |  |
|  | Primiparity | **1.28** | **1.09-1.49** | 1.08 | 0.83-1.42 |  | **1.31** | **1.12-1.53** | 1.01 | 0.78-1.31 |  | **1.53** | **1.31-1.80** | **0.67** | **0.51-0.86** |
|  | Multiparity | 1.00 | Reference | 1.00 | Reference |  | 1.00 | Reference | 1.00 | Reference |  | 1.00 | Reference | 1.00 | Reference |
| **Maternal smoking** | |  |  |  |  |  |  |  |  |  |  |  |  |  |  |
|  | Yes | **1.58** | **1.27-1.97** | 1.20 | 0.80-1.81 |  | **1.62** | **1.30-2.02** | 1.15 | 0.77-1.71 |  | **1.48** | **1.18-1.86** | **1.52** | **1.07-2.14** |
|  | No | 1.00 | Reference |  | Reference |  | 1.00 | Reference | 1.00 | Reference |  | 1.00 | Reference | 1.00 | Reference |
|  | *Not known* | 1.11 | 0.71-1.72 | 0.73 | 0.31-1.72 |  | 1.14 | 0.73-1.78 | 0.72 | 0.32-1.61 |  | 1.17 | 0.76-1.81 | 0.58 | 0.23-1.47 |
| **Maternal BMI** | |  |  |  |  |  |  |  |  |  |  |  |  |  |  |
|  | <18.5 | 1.53 | 0.90-2.59 | 1.51 | 0.61-3.74 |  | 1.14 | 0.63-2.08 | **2.73** | **1.36-5.49** |  | 1.39 | 0.79-2.42 | 1.91 | 0.88-4.17 |
|  | 18.5-24.9 | 1.00 | Reference | 1.00 | Reference |  | 1.00 | Reference | 1.00 | Reference |  | 1.00 | Reference | 1.00 | Reference |
|  | 25-29.9 | **1.45** | **1.21-174** | 1.27 | 0.93-1.74 |  | **1.28** | **1.06-1.54** | **1.81** | **1.35-2.42** |  | **1.37** | **1.14-1.65** | **1.50** | **1.12-2.02** |
|  | 30+ | **1.84** | **1.49-2.28** | **1.51** | **1.04-2.20** |  | **1.86** | **1.51-2.30** | 1.41 | 0.95-2.11 |  | **1.74** | **1.40 -2.16** | **1.82** | **1.28-2.56** |
|  | *Not known* | **1.76** | **1.30-2.38** | **1.83** | **1.12-3.00** |  | **1.58** | **1.15-2.15** | **2.42** | **1.53-3.82** |  | **1.75** | **1.29-2.37** | **1.83** | **1.12-2.99** |
| **Involuntary childlessness** | | |  |  |  |  |  |  |  |  |  |  |  |  |  |
|  | No (<2 years) | 1.00 | Reference | 1.00 | Reference |  | 1.00 | Reference | 1.00 | Reference |  | 1.00 | Reference | 1.00 | Reference |
|  | 2-4 years | 1.02 | 0.68-1.52 | 1.02 | 0.51-2.02 |  | 1.21 | 0.83-1.78 | 0.45 | 0.18-1.17 |  | 1.06 | 0.71-1.575 | 0.89 | 0.43-1.84 |
|  | 5 years or more | 1.10 | 0.54-2.25 | 1.21 | 0.40-3.66 |  | 1.09 | 0.54-2.22 | 1.25 | 0.40-3.86 |  | 1.24 | 0.63-2.41 | 0.75 | 0.17-3.26 |
| **Assisted reproduction** | | |  |  |  |  |  |  |  |  |  |  |  |  |  |
|  | Yes | 1.03 | 0.61-1.74 | 0.99 | 0.42-2.32 |  | 1.02 | 0.61-1.69 | 1.03 | 0.39-2.69 |  | 1.06 | 0.64-1.76 | 0.85 | 0.31-2.31 |
|  | No | 1.00 | Reference | 1.00 | Reference |  | 1.00 | Reference | 1.00 | Reference |  | 1.00 | Reference | 1.00 | Reference |

*Model including all evaluated class variables in the table. ^A^No confirmed ASD in first relatives or first cousins. ^B^Confirmed ASD in first relatives or in first cousins.
