# Supplementary material for: Perinatal and maternal factors associated with Autism Spectrum Disorder
Source: PLoS One. 2026 Mar 18;21(3):e0316968. doi: 10.1371/journal.pone.0316968 (PMC12998875; doi:10.1371/journal.pone.0316968)
Supplement: S6 Table — (DOCX) [file pone.0316968.s006.docx]

**Table S6. Children with ASD and controls, by presence of pregnancy, delivery complications, and infant characteristics.**

|  |  | | **Children with ASD N=996** | | **Controls N=9960** | |  | **Odds Ratio** | | **Adjusted* OR** | |
| --- | --- | --- | --- | --- | --- | --- | --- | --- | --- | --- | --- |
|  |  | | **N** | **(%)** | **n** | **(%)** |  | **OR** | **95% CI** | **OR** | **95% CI** |
| **Pregnancy complications/conditions** | | |  |  |  |  |  |  |  |  |  |
|  | Pre-eclampsia | | 43 | ( 4.3) | 346 | ( 3.5) |  | 1.25 | 0.91-1.73 | 1.11 | 0.80-1.54 |
|  | Diabetes type 1 | | 7 | ( 0.7) | 49 | ( 0.5) |  | 1.43 | 0.65-3.17 | 1.33 | 0.60-2.95 |
|  | Gestational diabetes | | 25 | ( 2.5) | 158 | ( 1.6) |  | **1.60** | **1.04-2.45** | 1.47 | 0.95-2.26 |
|  | Ablatio/bleeding pre partum | | 19 | ( 1.9) | 132 | ( 1.3) |  | 1.45 | 0.89-2.53 | 1.35 | 0.82-2.20 |
|  | Placenta previa | | 1 | ( 0.1) | 18 | ( 0.2) |  | 0.56 | 0.07-4.16 | - | - |
|  | Premature rupture of membranes | | 85 | ( 8.5) | 877 | ( 8.8) |  | 0.97 | 0.77-1.22 | 0.93 | 0.74-1.18 |
|  | Umbilical cord complications | | 6 | ( 0.6) | 38 | ( 0.4) |  | 1.58 | 0.67-3.75 | 1.58 | 0.66-3.77 |
|  | Maternal epilepsy | | 9 | ( 0.9) | 53 | ( 0.5) |  | 1.70 | 0.84-3.47 | 1.75 | 0.86-3.57 |
|  | Any of these | |  |  |  |  |  | **1.19** | **1.01-1.41** | 1.12 | 0.94-1.33 |
|  | None of these | | 814 | (81.7) | 8389 | (84.2) |  | 1.0 | Reference | 1.0 | Reference |
| **Start of delivery** | | |  |  |  |  |  |  |  |  |  |
|  | Induction | | 140 | (14.1) | 1100 | (11.0) |  | 1.22 | 0.95-1.55 | 1.19 | 0.93-1.52 |
|  | Spontaneous | | 775 | (77.8) | 8158 | (81.9) |  | 1.0 | Reference | 1.0 | Reference |
|  | Elective CS | | 83 | ( 8.3) | 708 | ( 7.1) |  | **1.34** | **1.11-1.62** | **1.22** | **1.01-1.48** |
| **Delivery mode in trial of labor^b^** | | |  |  |  |  |  |  |  |  |  |
|  | Vaginal delivery | | 694 | (69.7) | 7566 | (76.0) |  | 1.0 | Reference | 1.0 | Reference |
|  | Emergency CS | | 139 | (14.0) | 996 | (10.0) |  | **1.52** | **1.25-1.85** | **1.40** | **1.15-1.81** |
|  | Forceps/VE | | 80 | ( 8.0) | 690 | ( 6.9) |  | 1.26 | 0.99-1.61 | 1.22 | 0.95-1.57 |
| **Presentation** | | |  |  |  |  |  |  |  |  |  |
|  | Breech, other presentation | | 48 | ( 4.8) | 378 | ( 3.8) |  | 1.28 | 0.94-1.75 | 1.29 | 0.95-1.76 |
|  | Cephalic presentation | | 948 | (95.2) | 9582 | (96.2) |  | 1.0 | Reference | 1.0 | Reference |
| **Birth weight** | | |  |  |  |  |  |  |  |  |  |
|  | | <2500 | 59 | ( 5.9) | 429 | ( 4.3) |  | **1.39** | **1.05-1.84** | 1.28 | 0.96-1.70 |
|  | | 2500-4499 | 896 | (90.0) | 9076 | (91.1) |  | 1.0 | Reference | 1.0 | Reference |
|  | | >=4500 | 41 | ( 4.1) | 455 | ( 4.6) |  | 0.91 | 0.66-1.27 | 0.87 | 0.63-1.21 |
| **Growth** | |  |  |  |  |  |  |  |  |  |  |
|  | | SGA | 16 | ( 1.6) | 137 | ( 1.4) |  | 1.15 | 0.82-1.61 | 1.02 | 0.72-1.43 |
|  | | AGA | 868 | (87.1) | 8781 | (88.2) |  | 1.0 | Reference | 1.0 | Reference |
|  | | LGA | 112 | (11.2) | 1042 | (10.5) |  | 1.28 | 0.98-1.67 | 1.23 | 0.94-1.62 |
| **Apgar score 5 min** | | |  |  |  |  |  |  |  |  |  |
|  | | 0-6 | 20 | ( 2.0) | 164 | ( 1.6) |  | 1.22 | 0.77-1.96 | 1.09 | 0.68-1.74 |
|  | | 7-10 | 976 | (98.0) | 9796 | (98.4) |  | 1.0 | Reference | 1.0 | Reference |
| **Gestational age (w)** | | |  |  |  |  |  |  |  |  |  |
|  | | <32 | 14 | ( 1.4) | 93 | ( 0.9) |  | **1.79** | **1.03-3.12** | 1.66 | 0.95-2.92 |
|  | | 32-36 | 55 | ( 5.5) | 411 | ( 4.1) |  | 1.29 | 0.91-1.84 | 1.32 | 0.92-1.88 |
|  | | 37-41 | 812 | (81.5) | 8104 | (81.4) |  | 1.0 | Reference | 1.0 | Reference |
|  | | 42+ | 115 | (11.5) | 1352 | (13.6) |  | 1.01 | 0.79-1.30 | 1.08 | 0.84-1.39 |

*Adjusted for maternal age, parity, smoking, BMI, assisted reproduction, and involuntary childlessness. ^b^Elective cesarean section excluded
