# Supplementary material for: Perinatal and maternal factors associated with Autism Spectrum Disorder
Source: PLoS One. 2026 Mar 18;21(3):e0316968. doi: 10.1371/journal.pone.0316968 (PMC12998875; doi:10.1371/journal.pone.0316968)
Supplement: S7 Table — (DOCX) [file pone.0316968.s007.docx]

**Table s7. Autism Spectrum Disorder (ASD) severity, familial history, and presence of Intellectual Disability (ID), respectively, by pregnancy complications, delivery mode, and infant characteristics.**

|  |  | Mild ASD  N=754 | | Moderate/ severe ASD  N=242 | | ASD without ID N=737 | | ASD with ID  N=259 | | Non-familial ASD  N=724 | | Familial ASD N=150 | | Controls N=9960 | |
| --- | --- | --- | --- | --- | --- | --- | --- | --- | --- | --- | --- | --- | --- | --- | --- |
|  |  | n | ( % ) | n | ( % ) | n | ( % ) | n | ( % ) | n | ( % ) | n | ( % ) | n | ( % ) |
| Pregnancy / delivery | |  |  |  |  |  |  |  |  |  |  |  |  |  |  |
| Complications | |  |  |  |  |  |  |  |  |  |  |  |  |  |  |
|  | Pre-eclampsia | 33 | ( 4.4) | 10 | ( 4.1) | 31 | ( 4.2) | 12 | ( 4.6) | 31 | ( 4.3) | 7 | ( 4.7) | 346 | ( 3.5) |
|  | Diabetes type 1 | 5 | ( 0.7) | 2 | ( 0.8) | 6 | ( 0.8) | 1 | ( 0.4) | 5 | ( 0.7) | 1 | ( 0.7) | 49 | ( 0.5) |
|  | Gestational diabetes | 15 | ( 2.0) | 10 | ( 4.1) | 22 | ( 3.0) | 3 | ( 1.2) | 18 | ( 2.5) | 5 | ( 3.3) | 158 | ( 1.6) |
|  | Ablatio/bleeding pre partum | 17 | ( 2.3) | 2 | ( 0.8) | 17 | ( 2.3) | 2 | ( 0.8) | 13 | ( 1.8) | 5 | ( 3.3) | 132 | ( 1.3) |
|  | Placenta praevia | 1 | ( 0.1) | 0 | ( 0.0) | 1 | ( 0.1) | 0 | ( 0.0) | 0 | ( 0.0) | 1 | ( 0.7) | 18 | ( 0.2) |
|  | Premature rupture of membranes | 67 | ( 8.9) | 18 | ( 7.4) | 57 | ( 7.7) | 28 | (10.8) | 66 | ( 9.1) | 13 | ( 8.7) | 877 | ( 8.8) |
|  | Umbilical cord complications | 6 | ( 0.8) | 0 | ( 0.0) | 5 | ( 0.7) | 1 | ( 0.4) | 4 | ( 0.6) | 1 | ( 0.7) | 38 | ( 0.4) |
|  | Maternal epilepsy | 4 | ( 0.5) | 5 | ( 2.1) | 6 | ( 0.8) | 3 | ( 1.2) | 4 | ( 0.6) | 2 | ( 1.3) | 53 | ( 0.5) |
|  | Any of these | 138 | (18.3) | 44 | (18.2) | 135 | (18.3) | 47 | (18.1) | 132 | (18.2) | 31 | (20.7) | 1571 | (15.8) |
|  | None of these | 616 | (81.7) | 198 | (81.8) | 602 | (81.7) | 212 | (81.9) | 592 | (81.8) | 119 | (79.3) | 8389 | (84.2) |
| Starat of delivery | |  |  |  |  |  |  |  |  |  |  |  |  |  |  |
|  | Induction | 101 | (13.4) | 39 | (16.1) | 105 | (14.2) | 35 | (13.5) | 99 | (13.7) | 22 | (14.7) | 1100 | (11.0) |
|  | Spontaneous onset | 594 | (78.8) | 181 | (74.8) | 573 | (77.7) | 202 | (78.0) | 581 | (80.2) | 101 | (67.3) | 8158 | (81.9) |
|  | Elective CS | 60 | ( 8.0) | 23 | ( 9.5) | 60 | ( 8.1) | 23 | ( 8.9) | 46 | ( 6.4) | 27 | (18.0) | 708 | ( 7.1) |
| Delivery mode in trial of labor | |  |  |  |  |  |  |  |  |  |  |  |  |  |  |
|  | Vaginal birth, non instr | 530 | (70.3) | 164 | (67.8) | 513 | (69.6) | 181 | (69.9) | 510 | (70.4) | 93 | (62.0) | 7566 | (76.0) |
|  | Emergency CS | 105 | (13.9) | 34 | (14.0) | 103 | (14.0) | 36 | (13.9) | 101 | (14.0) | 24 | (16.0) | 996 | (10.0) |
|  | Forceps, VE | 59 | ( 7.8) | 21 | ( 8.7) | 61 | ( 8.3) | 19 | ( 7.3) | 67 | ( 9.3) | 6 | ( 4.0) | 690 | ( 6.9) |
| Presentation | |  |  |  |  |  |  |  |  |  |  |  |  |  |  |
|  | Breech/other presentation | 36 | ( 4.8) | 12 | ( 5.0) | 36 | ( 4.9) | 12 | ( 4.6) | 36 | ( 5.0) | 8 | ( 5.3) | 378 | ( 3.8) |
|  | Cephalic presentation | 718 | (95.2) | 230 | (95.0) | 701 | (95.1) | 247 | (95.4) | 688 | (95.0) | 142 | (94.7) | 9582 | (96.2) |
| Infant characteristics | |  |  |  |  |  |  |  |  |  |  |  |  |  |  |
| Birth weight | |  |  |  |  |  |  |  |  |  |  |  |  |  |  |
|  | BW<2550g | 49 | ( 6.5) | 10 | ( 4.1) | 38 | ( 5.2) | 21 | ( 8.1) | 46 | ( 6.4) | 7 | ( 4.7) | 429 | ( 4.3) |
|  | 2550g-4499g | 674 | (89.4) | 222 | (91.7) | 668 | (90.6) | 228 | (88.0) | 648 | (89.5) | 139 | (92.7) | 9076 | (91.1) |
|  | BW>=4500g | 31 | ( 4.1) | 10 | ( 4.1) | 31 | ( 4.2) | 10 | ( 3.9) | 30 | ( 4.1) | 4 | ( 2.7) | 455 | ( 4.6) |
| Growth | |  |  |  |  |  |  |  |  |  |  |  |  |  |  |
|  | SGA | 14 | ( 1.9) | 2 | ( 0.8) | 11 | ( 1.5) | 5 | ( 1.9) | 14 | ( 1.9) | 2 | ( 1.3) | 137 | ( 1.4) |
|  | AGA | 659 | (87.4) | 209 | (86.4) | 643 | (87.2) | 225 | (86.9) | 626 | (86.5) | 134 | (89.3) | 8781 | (88.2) |
|  | LGA | 81 | (10.7) | 31 | (12.8) | 83 | (11.3) | 29 | (11.2) | 84 | (11.6) | 14 | ( 9.3) | 1042 | (10.5) |
| Apr score 5 minutes | |  |  |  |  |  |  |  |  |  |  |  |  |  |  |
|  | Apg <7 | 16 | ( 2.1) | 4 | ( 1.7) | 15 | ( 2.0) | 5 | ( 1.9) | 17 | ( 2.3) | 2 | ( 1.3) | 164 | ( 1.6) |
|  | Apg 7+ | 738 | (97.9) | 238 | (98.3) | 722 | (98.0) | 254 | (98.1) | 707 | (97.7) | 148 | (98.7) | 9796 | (98.4) |
| Gestational age | |  |  |  |  |  |  |  |  |  |  |  |  |  |  |
|  | <32w | 15 | ( 2.0) | 2 | ( 0.8) | 10 | ( 1.4) | 7 | ( 2.7) | 11 | ( 1.5) | 3 | ( 2.0) | 101 | ( 1.0) |
|  | 32-36w | 51 | ( 6.8) | 16 | ( 6.6) | 46 | ( 6.2) | 21 | ( 8.1) | 52 | ( 7.2) | 9 | ( 6.0) | 536 | ( 5.4) |
|  | 37-41w | 637 | (84.5) | 202 | (83.5) | 628 | (85.2) | 211 | (81.5) | 609 | (84.1) | 125 | (83.3) | 8575 | (86.1) |
|  | 42+w | 50 | ( 6.6) | 22 | ( 9.1) | 52 | ( 7.1) | 20 | ( 7.7) | 51 | ( 7.0) | 13 | ( 8.7) | 745 | ( 7.5) |
