# Supplementary material for: Perinatal and maternal factors associated with Autism Spectrum Disorder
Source: PLoS One. 2026 Mar 18;21(3):e0316968. doi: 10.1371/journal.pone.0316968 (PMC12998875; doi:10.1371/journal.pone.0316968)
Supplement: S8 Table — (DOCX) [file pone.0316968.s008.docx]

**Table S8. Autism Spectrum Disorder (ASD) severity, familial history, and presence of Intellectual Disability (ID), respectively, by pregnancy complications, delivery mode, and infant characteristics.**

|  |  | **Mild ASD vs controls** | | **Moderate/Severe ASD vs controls** | | |  | | **ASD without ID vs controls** | | | **Autism with ID vs controls** | | |  | | **Non-familial^a^ ASD vs controls** | | | **Familial^b^ ASD vs controls** | | |  |
| --- | --- | --- | --- | --- | --- | --- | --- | --- | --- | --- | --- | --- | --- | --- | --- | --- | --- | --- | --- | --- | --- | --- | --- |
|  |  | **AOR^*^** | **95% CI** | | **AOR^*^** | **95% CI** | |  | | **AOR^*^** | **95% CI** | | **AOR^*^** | **95% CI** | |  | | **AOR^*^** | **95% CI** | | **AOR^*^** | **95% CI** | |
| **Pregnancy/delivery** | |  |  | |  |  | |  | |  |  | |  |  | |  | |  |  | |  |  | |
|  | Pre-eclampsia | 1.11 | 0.77-1.61 | | 1.09 | 0.57-2.08 | |  | | 1.05 | 0.72-1.54 | | 1.29 | 0.71-2.35 | |  | | 1.04 | 0.71-1.52 | | 1.32 | 0.73-2.40 | |
|  | Diabetes type 1 | 1.26 | 0.50-3.18 | | 1.52 | 0.36-6.36 | |  | | 1.54 | 0.65-3.64 | | 0.74 | 0.10-5.42 | |  | | 1.37 | 0.54-3.46 | | 1.23 | 0.30-5.14 | |
|  | Gestational diabetes | 1.20 | 0.70-2.05 | | **2.30** | **1.19-4.46** | |  | | **1.75** | **1.11-2.77** | | 0.67 | 0.21-2.13 | |  | | 1.47 | 0.89-2.42 | | 1.43 | 0.66-3.11 | |
|  | Ablatio/bleeding pre partum | 1.63 | 0.97-2.73 | | 0.54 | 0.13-2.23 | |  | | 1.61 | 0.96-2.71 | | 0.56 | 0.14-2.30 | |  | | 1.24 | 0.69-2.21 | | 1.65 | 0.72-3.81 | |
|  | Placenta previa | 0.77 | 0.10-5.80 | | - | - | |  | | 0.80 | 0.11-6.07 | | - | - | |  | | - | - | | 1.76 | 0.23-13.38 | |
|  | Premature rupture of membranes | 0.97 | 0.75-1.26 | | 0.80 | 0.49-1.31 | |  | | 0.83 | 0.62-1.09 | | 1.25 | 0.84-1.87 | |  | | 0.97 | 0.74-1.26 | | 0.82 | 0.51-1.32 | |
|  | Umbilical cord complications | 2.10 | 0.88-5.03 | | - | - | |  | | 1.70 | 0.66-4.37 | | 1.12 | 0.15-8.26 | |  | | 1.38 | 0.49-3.90 | | 2.19 | 0.52-9.23 | |
|  | Maternal epilepsy | 1.01 | 0.36-2.82 | | **4.11** | **1.62-10.41** | |  | | 1.54 | 0.66-3.62 | | 2.38 | 0.74-7.71 | |  | | 1.07 | 0.38-2.98 | | **3.59** | **1.41-9.14** | |
|  | Any of these | 1.12 | 0.93-1.37 | | 1.11 | 0.79-1.55 | |  | | 1.11 | 0.91-1.35 | | 1.16 | 0.84-1.61 | |  | | 1.08 | 0.89-1.32 | | 1.23 | 0.89-1.68 | |
|  | None of these | 1.00 | Reference | | 1.00 | Reference | |  | | 1.00 | Reference | | 1.00 | Reference | |  | | 1.00 | Reference | | 1.00 | Reference | |
| **Start of delivery** | |  |  | |  |  | |  | |  |  | |  |  | |  | |  |  | |  |  | |
|  | Induction | 1.18 | 0.89-1.56 | | 1.25 | 0.79-1.98 | |  | | 1.20 | 0.90-1.59 | | 1.19 | 0.76-1.88 | |  | | 0.89 | 0.64-1.22 | | **2.03** | **1.41-2.94** | |
|  | Spontaneous | 1.00 | Reference | | 1.00 | Reference | |  | | 1.00 | Reference | | 1.00 | Reference | |  | |  | Reference | |  | Reference | |
|  | Elective CS | 1.15 | 0.92-1.44 | | **1.46** | **1.02-2.09** | |  | | 1.23 | 0.99-1.53 | | 1.21 | 0.84-1.74 | |  | | 1.14 | 0.91-1.43 | | **1.47** | **1.04-2.09** | |
| **Delivery mode in trial of labor^c^** | |  |  | |  |  | |  | |  |  | |  |  | |  | |  |  | |  |  | |
|  | Vaginal delivery | 1.00 | Reference | | 1.00 | Reference | |  | | 1.00 | Reference | | 1.00 | Reference | |  | | 1.00 | Reference | | 1.00 | Reference | |
|  | Emergency CS | **1.39** | **1.11-1.74** | | 1.45 | 0.98-2.13 | |  | | **1.38** | **1.10-1.73** | | **1.48** | **1.02-2.15** | |  | | **1.34** | **1.06-1.68** | | **2.01** | **1.39-2.91** | |
|  | Forceps/VE | 1.16 | 0.87-1.55 | | 1.43 | 0.89-2.29 | |  | | 1.22 | 0.92-1.62 | | 1.21 | 0.74-1.97 | |  | | 1.29 | 0.98-1.69 | | 0.94 | 0.52-1.67 | |
| **Presentation** | |  |  | |  |  | |  | |  |  | |  |  | |  | |  |  | |  |  | |
|  | Breech, other presentation | 1.28 | 0.90-1.82 | | 1.32 | 0.73-2.39 | |  | | 1.31 | 0.92-1.86 | | 1.25 | 0.69-2.25 | |  | | 1.30 | 0.91-1.85 | | 1.29 | 0.71-2.33 | |
|  | Cephalic presentation | 1.00 | Reference | | 1.00 | Reference | |  | | 1.00 | Reference | |  | Reference | |  | | 1.00 | Reference | | 1.00 | Reference | |
| **Infant characteristics** | |  |  | |  |  | |  | |  |  | |  |  | |  | |  |  | |  |  | |
| **Birth weight** | |  |  | |  |  | |  | |  |  | |  |  | |  | |  |  | |  |  | |
|  | <2500 | **1.41** | **1.03-1.92** | | 0.87 | 0.46-1.67 | |  | | 1.09 | 0.77-1.53 | | **1.87** | **1.18-2.98** | |  | | 1.35 | 0.98-1.86 | | 1.07 | 0.61-1.90 | |
|  | 2500-4499 | 1.00 | Reference | | 1.00 | Reference | |  | | 1.00 | Reference | | 1.00 | Reference | |  | | 1.00 | Reference | | 1.00 | Reference | |
|  | >=4500 | 0.88 | 0.60-1.28 | | 0.85 | 0.45-1.62 | |  | | 0.89 | 0.61-1.29 | | 0.83 | 0.44-1.58 | |  | | 0.91 | 0.62-1.34 | | 0.78 | 0.42-1.44 | |
| **Growth** | |  |  | |  |  | |  | |  |  | |  |  | |  | |  |  | |  |  | |
|  | SGA | 1.06 | 0.73-1.55 | | 0.87 | 0.42-1.78 | |  | | 1.00 | 0.68-1.49 | | 1.07 | 0.56-2.05 | |  | | 1.14 | 0.78-1.65 | | 0.69 | 0.32-1.48 | |
|  | AGA | 1.00 | Reference | | 1.0 | Reference | |  | | 1.00 | Reference | | 1.00 | Reference | |  | | 1.00 | Reference | | 1.00 | Reference | |
|  | LGA | 1.23 | 0.90-1.68 | | 1.25 | 0.74-2.10 | |  | | 1.23 | 0.90-1.69 | | 1.23 | 0.74-2.04 | |  | | 1.34 | 0.98-1.83 | | 1.00 | 0.59-1.68 | |
| **Apgar score 5 min** | |  |  | |  |  | |  | |  |  | |  |  | |  | |  |  | |  |  | |
|  | 0-6 | 1.14 | 0.67-1.92 | | 0.94 | 0.34-2.57 | |  | | 1.08 | 0.63-1.84 | | 1.13 | 0.46-2.79 | |  | | 1.21 | 0.73-2.02 | | 0.68 | 0.21-2.15 | |
|  | 7-10 | 1.00 | Reference | | 1.00 | Reference | |  | | 1.00 | Reference | | 1.00 | Reference | |  | | 1.00 | Reference | | 1.00 | Reference | |
| **Gestational age (w)** | |  |  | |  |  | |  | |  |  | |  |  | |  | |  |  | |  |  | |
|  | <32 | **2.12** | **1.16-3.89** | | 0.62 | 0.14-2.68 | |  | | 1.41 | 0.71-2.80 | | 2.37 | 0.97-5.78 | |  | | 1.56 | 0.80-3.04 | | 1.97 | 0.77-5.04 | |
|  | 32-36 | 1.46 | 0.97-2.19 | | 1.01 | 0.52-1.94 | |  | | 1.26 | 0.83-1.91 | | 1.45 | 0.77-2.71 | |  | | 1.47 | 0.98-2.20 | | 0.97 | 0.49-1.91 | |
|  | 37-41 | 1.00 | Reference | | 1.00 | Reference | |  | | 1.00 | Reference | | 1.00 | Reference | |  | | 1.00 | Reference | | 1.00 | Reference | |
|  | 42+ | 1.20 | 0.87-1.61 | | 0.82 | 0.52-1.29 | |  | | 1.15 | 0.85-1.54 | | 0.92 | 0.58-1.47 | |  | | 1.14 | 0.85-1.54 | | 0.92 | 0.58-1.46 | |

*Adjusted for maternal age, parity, smoking, BMI, assisted reproduction, and involuntary childlessness. ^a^No confirmed ASD in first relatives or first cousins. ^b^Confirmed ASD in first relatives or in first cousins. ^c^Elective cesarean section excluded.
