# Supplementary material for: Perinatal and maternal factors associated with Autism Spectrum Disorder
Source: PLoS One. 2026 Mar 18;21(3):e0316968. doi: 10.1371/journal.pone.0316968 (PMC12998875; doi:10.1371/journal.pone.0316968)
Supplement: Attachment — Submitted filename: Response to reviewers.docx [file pone.0316968.s010.docx]

 PONE-D-24-58795

Perinatal and maternal factors associated with Autism Spectrum Disorder.

*Journal Requirements:*

*When submitting your revision, we need you to address these additional requirements.*

1. *Please ensure that your manuscript meets PLOS ONE's style requirements, including those for file naming. The PLOS ONE style templates can be found at*
   [*https://journals.plos.org/plosone/s/file?id=wjVg/PLOSOne_formatting_sample_main_body.pdf*](https://journals.plos.org/plosone/s/file?id=wjVg/PLOSOne_formatting_sample_main_body.pdf) *and* 
   [*https://journals.plos.org/plosone/s/file?id=ba62/PLOSOne_formatting_sample_title_authors_affiliations.pdf*](https://journals.plos.org/plosone/s/file?id=ba62/PLOSOne_formatting_sample_title_authors_affiliations.pdf)

We have checked that the manuscript meets with PLOS ONE´s style requirements.

2. *Thank you for stating the following financial disclosure: This study was supported by grant R01ES026904 from the National Institute of Environmental Health Sciences (NIEHS). The study was also supported by The Beatrice and Samuel L Seaver foundation (AR and AK).
   Please state what role the funders took in the study.  If the funders had no role, please state: ""The funders had no role in study design, data collection and analysis, decision to publish, or preparation of the manuscript."" 
   If this statement is not correct you must amend it as needed.*

*Please include this amended Role of Funder statement in your cover letter; we will change the online submission form on your behalf.*

We have added this statement in our cover letter.

3. *In the online submission form, you indicated that the register data are not publicly available due to privacy protection, including General Data Protection Regulations (GDPR). Access to Swedish register resources is only granted after ethical review by appropriate authorities. Requests should be directed to registrator@lu.se.
   All PLOS journals now require all data underlying the findings described in their manuscript to be freely available to other researchers, either 1. In a public repository, 2. Within the manuscript itself, or 3. Uploaded as supplementary information.
   This policy applies to all data except where public deposition would breach compliance with the protocol approved by your research ethics board. If your data cannot be made publicly available for ethical or legal reasons (e.g., public availability would compromise patient privacy), please explain your reasons on resubmission and your exemption request will be escalated for approval.*

Data cannot be shared publicly because register data are not publicly available due to privacy protection, including General Data Protection Regulation (GDPR). Access to Swedish register resources is only granted after ethical review by appropriate authorities. Requests should be directed to registrator@lu.se.

4. *We note that you have included the phrase “data not shown” in your manuscript. Unfortunately, this does not meet our data sharing requirements. PLOS does not permit references to inaccessible data. We require that authors provide all relevant data within the paper, Supporting Information files, or in an acceptable, public repository. Please add a citation to support this phrase or upload the data that corresponds with these findings to a stable repository (such as Figshare or Dryad) and provide and URLs, DOIs, or accession numbers that may be used to access these data. Or, if the data are not a core part of the research being presented in your study, we ask that you remove the phrase that refers to these data.*

We have added a supplementary table showing the numbers that were previously referred to in text.

**Comments to the Author**

*Reviewer #1: Overview:
This paper utilises comprehensive population-based data with validated diagnostic variables in an attempt to provide an overview of maternal and perinatal risk factors for ASD. It is commendable in its thoroughness in identifying rpotential risk factors and carrying out an extensive sets of analyses.
However, it is the reviewer’s opinion that the manuscript in its current form suffers from a number of significant shortcomings in terms of methodological problems, unclear or erroneous presentation of currently available evidence, methods, and results. Large-scale revising including redo of the main analyses with appropriate statistical models are needed before it may be deemed publishable. Comments are categorsied by sections as below:*

We thank this referee for the extensive and thorough review. We have done our best to meet each question and concern in our answers and we have also adjusted the manuscript accordingly.

*ABSTRACT
1. Abstract needs to be reformulated. There are texts that offered little information (for example, ‘following thorough review of medical records’), or are confusing out of context (for example, the mention of cases being ‘classified based on level of intellectual disability’ without explaining the relevance of intellectual disability first). Findings other than those concerning maternal weight (for example, regarding maternal smoking and delivery mode) were not described.*

We have revised the Abstract to provide a more balanced summary of our findings, in line with the reviewer’s suggestion. In addition to the association between maternal BMI and ASD, the Abstract now highlights other significant results, including maternal smoking, cesarean delivery, advanced maternal age, gestational diabetes, maternal epilepsy, and low Apgar scores (i.e., both shared and subgroup-specific associations). Phrases offering little standalone information (e.g., “following thorough review of medical records”) have been removed or rephrased for clarity and conciseness. The presentation of case classification has been simplified to avoid introducing subgroup definitions (e.g., intellectual disability) without prior context. The revised Abstract now provides a concise yet comprehensive summary of the study design, analytical approach, and principal results.

*INTRODUCTION
2. Introduction: overall, this section is too short and fails to cover crucial grounds. It should starts with a description of the disease entity (it's epidemiology, clinical presentation, burden etc) and goes on to discuss available evidence relevant to the research question(s) of this study. The section usually concludes with an exposition of the aims and objectives of the study.*

We have revised the manuscript in accordance with the reviewer’s comment. The Introduction has been expanded as recommended, now including information on epidemiology, clinical presentation and burden.

*2.a. The discussion on available evidence on maternal and perinatal risk factors for ASD, the grand theme of the current manuscript, is very scarce (a mere paragraph of one sentence). In-depth description of strength of evidence, important latest available studies (large-scale longitudinal studies or systematic reviews such as Hisle-Gorman et al. 2018), gaps in the current evidence base relevant to your paper should be described.*

In the revised manuscript, we have considerably extended the discussion on maternal and perinatal risk factors for ASD. The section now includes a broader overview of recent large-scale studies and systematic reviews, as well as a more detailed consideration of the consistency and limitations of the existing evidence in relation to our findings.

*2.b. The discussion on ASD+ID is necessary as ASD with or without ID is analysed separately later on, but the importance of doing so is not properly established here. Which ‘worse outcomes’ are associated with ID comorbidity? Of the many comorbidities that leads to poorer outcomes for*

We have added a discussion of relevant research on maternal and perinatal risk factors for ASD. The rationale for analyzing intellectual disability (ID) separately is now explicitly stated.

*2.c. There is insufficient discussion on the effect of familial history.*

The role of familial history is described.

*2.d. p3: '… environmental factors are also likely to play a role': which environmental factors?*

We have included reference to environmental factors relevant to ASD.

*2.e. p3: '… during a critical period in pregnancy': more details are needed. Which period specifically is critical for ASD development and how is this relevant under the given context?*

We now describe the critical period of fetal brain development during pregnancy.

*2.f. The larger half of the last paragraph (from ‘Information regarding factors associated with pregnancy…’) should be in Methods instead.*

Methodological details that were previously in the last paragraph of the Introduction have been moved to the Methods section.

2 a-f Additional references have been incorporated to support the reviewers 2a-f points.

*METHODS
3. Methods: This section failed to present the methodologies clearly and led the reviewer uncertain if appropriate methods have been used, most notably regarding samplig, statistical model specification, and variable descrioptions/definitions.*

*3.a. p5: The Skania Autism Study, the larger project on which the current paper is part of, needs more descriptions e.g. data collection, sample size, demographics, length of follow-up, attrition rate etc.*

We have revised the *Methods section, first and second paragraphs* to clarify the description of the Skania Autism Study (SAS), specifying that it is a total population-based ASD cohort with linked national and regional registers.

*3.b. p5: Youth- and habilitation center is not a familiar establishment for readers outside Sweden. A brief introduction would be helpful. Also there are mentions of similar terms like ‘habilitation clinic’ elsewhere. If they are one and the same the terminology should be standardised.*

The term “habilitation clinic” is now explained as a free, voluntary service to which most children with autism in the region are referred after diagnosis.

*3.c. p5: the abbreviation ID (here presumably for identification) is confusing as the same abbreviation has been defined and otherwise used in this manuscript for intellectual disability.*

The abbreviation “ID” is now used exclusively for “intellectual disability.”

*3.d. p6: it is not mentioned if any data derived from the blood samples were used in the current study. If these are not relevant, the blood sample analyses should not be mentioned in the Methods section.*

Irrelevant details on biosample procedures have been removed, while retaining essential contextual information. (The reason to why we only had one control for each case, was that those chemical analyses are expensive, and the study finances did not allow us to analyse 10 control samples for each case sample. However, in the revised version, we refrain from describing the sample procedure).

*3.e. p7: it is not randomly selected if it is frequency-matched.*

The control selection process is described in detail, including that controls were randomly selected from the general population within strata of birth year and sex.

*3.f. p7: what is the rationale of selecting one (and only one) control for the same type of blood analyses? As discussed, if the blood analyses are irrelevant to the current study this should not be described*

The reason to why we only had one control for each case, was that those chemical analyses are expensive, and the study finances did not allow us to analyse 10 control samples for each case samle. However, in the revised version, we refrain from describing the sample procedure.

*3.g. p8: for a matched case-control study, conditional logistic regression is the appropriate statistical model to account for the clustered structure of the data.*

Regarding the choice of statistic method, a conditional logistic regression model can be justified if the assumption that the cases and controls are similar holds– with the exception of the status (ASD in our case). That requires that the cases and controls are similar on many background variables. If (which we have not) such a thorough matching procedure is carried out, it is not possible to evaluate any of the factors that were used in the matching procedure. In our case, it would have meant that we had matched on all these risk factors that we wanted to investigate. We would have found nothing… Our controls were not thoroughly matched, but we wanted the male/female proportion, and year of birth to be similar between the study groups (meaning that those two factors could not be included in the analyses).

*3.h. p8: For most of the variables described here, how the data are collected failed to be sufficiently described. For example, when was maternal maternal smoking and BMI measured? How was involuntary childlessness defined?*

The timing and method of data collection for maternal characteristics, including BMI, years of involuntary childlessness, and smoking, are now clearly stated as self-reported at the first antenatal visit (approximately 9–12 gestational weeks).

Furthermore, the sentence on biosample collection has been relocated within the Methods for improved flow.

*RESULTS
4. Results: Here a number of statistical terms are used in unclear or erroneous manners.
4.a. p15: 'significantly increased ORs' is an incorrect way to describe the results. OR (odds ratio) is already a comparison between (the odds) of two groups.*

We have revised the Results section, multiple paragraphs - to replace the phrase “significantly increased ORs” with “higher odds” or equivalent wording.

*4.b. p15: ‘Maternal smoking was a significant factor associated with ASD’: this is confusing, is it significantly associated or is it being qualified as significant for other, unspecified, reasons?*

*And
4.c. p15 ‘Maternal smoking was a significant factor associated with ASD, for all ASD subgroups’: as far as I can tell from the tables, this is not true for moderate/severe ASD.*

The description of maternal smoking results is now precise, stating that maternal smoking during pregnancy was associated with mild ASD across all subgroups—that is, among children with and without intellectual disability, and with and without reported familial ASD—and was also associated with moderate/severe familial ASD.

*4.d. p15: '... A u-shaped association between ASD and BMI was indicated, but far from statistically significant(P>0.9)': It is unclear what statistical test was performed to test the for this u-shaped association. Once again, 'maternal BMI' should be specified to avoid confusion.*

The test for a U-shaped association between BMI and ASD has been specified as a quadratic term (p ≈ 0.880), and the timing of BMI measurement has been clarified as self-reported at the first antenatal visit (approximately 9–12 gestational weeks.

*DISCUSSION
5.a. p24: ‘In our study, information on smoking were obtained during the first trimester’: this information should be in the Methods section.*

The statement on the timing of maternal smoking data collection has been moved from the Discussion to the Methods section, where it is now described as self-reported at the first antenatal visit (approximately 9–12 gestational weeks).

*5.b. p26: Why and how may paternal age be an important confounder? There should be more discussion on this.*

The discussion of paternal age as a potential confounder has been expanded including biological mechanisms (reduced sperm quality, de novo mutations, epigenetic alterations) and the combined influence of maternal and paternal age on ASD risk.

*5.c. p28: Discussions on strengths and limitations are insufficient. Take limitations as the example, the only one discussed was a lack of access to certain variables. What limitations may have been incurred from the study design, analytical/statistical models, data collection and variable definition, etc?*

We have also expanded the *Strengths and Limitations section* to include potential residual confounding, particularly for socioeconomic factors; possible misclassification in self-reported exposures such as smoking and BMI; and variability in ASD diagnosis. We have also discussed the generalizability of the findings, noting that they apply primarily to the Swedish healthcare context and may not directly transfer to populations with different healthcare systems or demographic profiles. The conditional logistic regression models assume proportional odds and may be sensitive to model specification, which could influence the estimated associations. In addition, some variable definitions (e.g., exposure categories for maternal BMI and smoking) are based on self-reported or register-derived data, which may lead to misclassification and attenuated associations.

*5.d. p29: More discussion on the policy/clinical/real-world implications is needed.*

The implications of our findings for policy and clinical practice are mentioned in the final part of the Discussion, noting that the results may inform earlier identification strategies and support the development of more personalized clinical guidelines.

We have revised the *Discussion section, multiple paragraphs* to integrate our findings with a broader range of previous literature, including large cohort studies and meta-analyses. Differences across ASD subgroups are explicitly described, highlighting where associations were stronger for mild ASD or ASD without intellectual disability. We have also included specific examples from our results tables to illustrate subgroup differences. The sequence in which findings are presented in the Discussion has been revised ensuring a more logical flow. For greater readability, each variable (risk factor) is now discussed under its own subheading, making it easier for the reader to follow and locate specific findings within the expanded Discussion. These changes were undertaken to enhance the transparency and accessibility of the Discussion section, particularly in light of its increased length following the incorporation of additional literature and subgroup analyses.

*6. Language: Beyond those mentioned above, a large number of other language and editing problems renders the manuscript difficult to understand and ineffective in conveying the scientific information. It also gives the manuscript the look of an unfinished document. The manuscript needs to undergo extensive and professional English editing if not extensive rewriting. Examples are given in the following pointers but they are not meant to be exhaustive:*

*6.a. grammar: a comma is missing after ‘compared with ASD with ID’ ( in Introduction, p3); ‘First data from habilitation register and perinatal register accessed 2017-09-25’ is not a complete sentence (Methods, Ethics)*

*6.b. typo: chronic instead of chronical diseases (Methods, p5)*

*6.c. clarity: some sentences are difficult to comprehend. For example,’including children to mothers who were born in Sweden only’ (Introduction, p4) is confusing; ‘The medical records from the habilitation clinics were particularly useful…’: in what way useful? specifically how are these medical records used? Or instead, simply ‘The medical records from the habilitation clinics were used to assess level of ASD severity’ if those records were indeed the sole data used for severity triage; overweight and obesity were two terms used in this manuscript for several times but never defined; still another example is the missing ‘maternal’ where it is presumably needed (e,g. Resultsm ‘Furthermore, a strong and significant association between increasing BMI and ASD was found’);*

*6.d. incorrect word choice: Introduction, p3, (‘Swedish national clinical guidelines assume’) a better verb would be ‘estimate’; Methods p7: strong impression instead of strong hypothesis; Discussion, p26: ‘heterogenic relationships in Sweden’ is confusing. Do you mean heterosexual?
6.e. scientific writing quality: some sentences need to be rewritten to be more informativ, more succinct, or both. For example, in Introduction, p3 (Comorbidity is common and many individuals with ASD face an increased risk of physical and psychological health problems.), the second clause adds little new information to the first. May rewrite it into “Individuals with ASD face an increased risk of physical and psychological health problems, including………”; Discussion, p24: ‘The results are in line with von Ehrenstein et al., who…’: not in line with the researcher, but with their findings or with the paper. In either case the sentence needs rework; Discussion, p25: ‘probably several would follow this advice’ reads conjectural and should be backed by evidence or sounder arguments.
6.f. other English editing issues: unstandardised indentations (e.g. Introduction, paragraph 1 vs the rest); unwarranted shade behind texts (e.g. in Introduction, p5)*

6a-6f: We appreciate the reviewer’s detailed feedback on the language and clarity of the manuscript. Since the original submission, the manuscript has undergone a comprehensive, section-by-section revision to improve the precision, grammar, and readability of the text. All sentences have been reviewed and rewritten where needed to ensure clarity and alignment with academic English standards. The examples highlighted by the reviewer have either been corrected directly or are no longer applicable due to substantial rewriting. In addition, formatting inconsistencies such as indentation and background shading have been corrected.

*Reviewer #2: This study examined maternal and perinatal conditions associated with ASD, integrating population-based registry data with case-level diagnostic validation to enhance reliability. The findings indicate that maternal overweight/obesity is a significant risk factor for ASD, suggesting modifiable maternal health factors that may inform prevention strategies. These findings provide valuable insights into potential ASD risk factors. However, one concern warrants further discussion.

There are several notable findings related to familial ASD, including higher maternal age, elective C-section, and maternal epilepsy, which were associated specifically with familial ASD but not non-familial ASD. To better understand whether these associations are influenced by maternal-specific factors, paternal contributions, or broader familial genetics, further analysis distinguishing maternal vs. paternal familial ASD should be considered. This distinction could help determine whether these associations result from confounding factors, such as parental characteristics or reproductive patterns, or if they reflect genuine interactions between environmental and genetic influences.*

We thank the reviewer for highlighting the importance of distinguishing between maternal and paternal lineage in the definition of familial ASD. We agree that this distinction could provide valuable insights into whether associations observed in the familial subgroup reflect predominantly maternal factors, paternal factors, or shared genetic influences. In response, we have expanded the *Strengths and Limitations* section to explicitly acknowledge the lack of lineage-specific information as a limitation of the current study and to note that this precludes further interpretation of associations such as those with higher maternal age, elective cesarean section, and maternal epilepsy. We have also suggested that future studies incorporate detailed genealogical and clinical data from both maternal and paternal lineages to enable a more precise evaluation of these relationships.

Note on references
Due to the extensive rewriting and restructuring of the manuscript, many in-text citation numbers have changed, although the majority of references remain the same as in the original submission. Several new references have been added to strengthen the coverage of recent and relevant literature while some older references were removed as they were no longer directly relevant.

*While revising your submission, please upload your figure files to the Preflight Analysis and Conversion Engine (PACE) digital diagnostic tool,* [*https://pacev2.apexcovantage.com/*](https://track.editorialmanager.com/CL0/https:%2F%2Fpacev2.apexcovantage.com%2F/1/010f01954dffe7c8-11e4526b-906c-472b-9004-dcd5c5a87bfc-000000/F1f87zsFjlNGW5HpA_MvCNoPztmaZ77WIgPvMvHZ6M8=200)*. PACE helps ensure that figures meet PLOS requirements. To use PACE, you must first register as a user. Registration is free. Then, login and navigate to the UPLOAD tab, where you will find detailed instructions on how to use the tool. If you encounter any issues or have any questions when using PACE, please email PLOS at* [*figures@plos.org*](mailto:figures@plos.org)*. Please note that Supporting Information files do not need this step.*

We have used the PACE tool to ensure that our figure meets PLOS’s requirements.
