# Supplementary material for: Perinatal and maternal factors associated with Autism Spectrum Disorder
Source: PLoS One. 2026 Mar 18;21(3):e0316968. doi: 10.1371/journal.pone.0316968 (PMC12998875; doi:10.1371/journal.pone.0316968)
Supplement: Attachment — Submitted filename: Rebuttal letter.docx [file pone.0316968.s011.docx]

**Dear Editor,**

We thank you and the reviewers for the careful and constructive evaluation of our manuscript. We have revised the manuscript in response to all comments raised during the review process. Below, we provide a detailed, point-by-point response describing how each comment has been addressed in the revised version.

**Editorial comment on wording and phrasing**

As specifically requested by the Academic Editor, Dr. Mu-Hong Chen, we carefully reviewed the wording and phrasing throughout the entire manuscript to ensure that the language is appropriate and not potentially harmful or offensive to the autistic community. In particular, we addressed concerns regarding wording that could be interpreted as implying prevention of autism.

In the revised manuscript, we clarified that terms such as *“risk”* and *“association”* are used strictly in an epidemiological, non-normative sense, consistent with the observational design of the study. Specifically, we revised wording that could be construed as prevention-oriented and replaced it with language emphasizing etiological understanding, subgroup heterogeneity, and how maternal and perinatal health contexts relate to neurodevelopmental diversity. The revised text is framed within a neurodiversity-informed and epidemiological perspective and does not suggest preventive goals.

**Responses to Reviewers**

**Reviewer #1**

We thank Reviewer #1 for the overall positive assessment of the revised manuscript and for the helpful suggestions.

**Comment 1: Standardize terminology related to sex and gender**

**Response:**
We have revised the entire manuscript, including all tables and figure captions, to ensure consistent terminology. We now use *sex* throughout when referring to biological classification and consistently refer to *males* and *females* rather than age-specific terms such as *boys* and *girls*. References to gender have been removed to avoid ambiguity.

**Comment 2: Definition and terminology of familial vs. non-familial ASD**

**Response:**
We have clarified and made explicit the definition of familial versus non-familial ASD in the Methods section. Specifically, cases are now defined as *familial ASD* if at least one first- or second-degree relative had a documented ASD diagnosis, while *non-familial ASD* refers to the absence of any reported ASD diagnosis among close relatives rather than missing information.
In addition, terminology has been harmonized throughout the manuscript and tables by consistently using the terms *family history*, *familial ASD* and *non-familial ASD*, replacing the previously used term *heredity*.

**Comment 3: Use of conditional vs. unconditional logistic regression**

**Response:**
We thank the reviewer for raising this important methodological point. We maintain that unconditional logistic regression was the most appropriate approach for the present study design. A conditional logistic regression model can be justified when cases and controls are closely matched on multiple background variables, apart from outcome status. In our study, such extensive matching was not performed, as it would have precluded evaluation of several exposures of interest. Instead, we applied a loose-matching design, frequency-matching cases and controls on sex and year of birth only. Accordingly, we follow the reviewer’s alternative 3c. We have added a paragraph in the Discussion addressing this methodological choice and citing relevant methodological literature on the performance of conditional versus unconditional logistic regression in loosely matched data. In addition, we have clarified the matching strategy and justification for unconditional logistic regression in the Methods section, supported by methodological evidence indicating that this approach yields valid estimates when matching variables and relevant covariates are included (Kuo et al., 2018). Finally, we corrected an erroneous statement in the abstract regarding the use of conditional logistic regression, which arose due to a misunderstanding between authors.

**Reviewer #2**

**Response:**
We thank Reviewer #2 for the positive evaluation and for confirming that all comments have been addressed.

**Reviewer #3**

We thank Reviewer #3 for the detailed and constructive comments.

**Comment 1–2: Novelty relative to prior Scandinavian registry studies**

**Response:**
We agree that the novelty of our study required clearer articulation. We have therefore added a new section entitled *“Novelty and Contribution”* immediately before the Strengths and Limitations section. This section clarifies how our study extends prior Nordic register research by using individually confirmed DSM-5 diagnoses, restricting analyses to mothers born in Sweden to reduce heterogeneity related to migration and ethnicity, and examining subgroup-specific association patterns by ASD severity, intellectual disability, and familial ASD. A brief reference to a recent large Nordic register study has also been added to the Discussion to contextualize our findings.

**Comment 3: Rationale for subgroup stratification**

**Response:**
We added a short explanatory paragraph in the Introduction clarifying the rationale for stratifying by ASD severity, intellectual disability, and familial ASD. The text explains that these dimensions capture both clinical and etiological heterogeneity, as severity and intellectual disability have been linked to partly distinct neurodevelopmental and genetic mechanisms, while familial ASD reflects inherited liability that may modify environmental associations.

**Comment 4: Power limitations and multiple comparisons**

**Response:**
We have revised the Strengths and Limitations section to explicitly address potential power limitations in smaller subgroup strata, particularly severe ASD and ASD with co-occurring intellectual disability, where sample sizes were limited and confidence intervals wider. We now clarify that subgroup-specific estimates should therefore be interpreted with caution. We also explicitly acknowledge that the large number of subgroup analyses increases the risk of chance findings due to multiple comparisons. Accordingly, we emphasize interpretation based on effect sizes, confidence intervals, and consistency of patterns across related analyses rather than on statistical significance alone.

**Comment 5: Advanced maternal age and heritable traits**

**Response:**
We expanded the conceptual discussion in the Maternal Age section to clarify that associations between advanced maternal age and ASD, particularly in the familial subgroup, may reflect shared heritable traits and assortative mating rather than intrauterine causal mechanisms. Relevant references were added to support this interpretation.

**Comment 6: Collinearity among maternal variables**

**Response:**
Maternal BMI, smoking, and diabetes were included simultaneously in the multivariable models. While we did not perform a formal collinearity diagnostics, we did not observe indications of model instability or implausible coefficient estimates when these variables were entered together. We therefore consider the risk of problematic collinearity to be limited, but acknowledge this as a potential methodological consideration.

**Comment 7: Justification for variable inclusion**

**Response:**
Variables were included based on prior epidemiological evidence and data availability rather than statistical selection procedures. Specifically, included factors were those that have previously been associated with ASD or with pregnancy and delivery complications, as now explicitly stated in the Statistical Analyses section.

**Comment 8: Paternal age**

**Response:**
We agree that paternal age may be an important confounder, given its correlation with maternal age and its established association with ASD risk. Unfortunately, information on paternal age was not available and could therefore not be included in sensitivity analyses. As discussed in the manuscript, maternal and paternal ages are strongly correlated in Sweden, and observed associations with maternal age likely reflect shared parental age-related and familial factors. This limitation is explicitly acknowledged.

**Comment 9: Missing BMI data**

**Response:**
Missing BMI values were present in 8.3% of ASD cases and 5.8% of controls, suggesting that missingness was not random. Rather than applying imputation methods that assume data are missing at random, we therefore treated missing BMI as a separate category. As discussed in the Discussion, we suspect that non-response may be related to underlying maternal characteristics, including overweight or obesity. To assess robustness, we also performed complete-case analyses; estimates for the other maternal covariates changed only marginally compared with the main analyses, indicating that the overall findings were not driven by the handling of missing BMI data. We note that the study was conducted within a single regional public healthcare system with standardized antenatal care routines, making systematic hospital-level differences in BMI recording unlikely.

**Comment 10: Cesarean delivery**

**Response:**
We added a statement in the Discussion noting that associations between cesarean delivery and ASD may reflect confounding by indication rather than procedure-specific effects, supported by large meta-analyses and sibling-based studies.

**Comment 11: Smoking data**

**Response:**
We clarified that smoking was assessed only at the first antenatal visit and that later changes in smoking behavior could not be captured. This limitation is now explicitly discussed, along with the potential for residual confounding by socioeconomic and maternal mental health factors.

**Comment 12: Mechanistic discussion**

**Response:**
A brief statement was added noting that future studies integrating biological data may help clarify underlying mechanisms.

**Comments 13–15: Tables and figures**

**Response:**
Supplementary tables were expanded to include absolute numbers and total subgroup sizes. Key findings are now summarized using forest plots to improve readability, and presentation of adjusted odds ratios and confidence intervals has been standardized.

**Minor comments and formatting**

Minor typographical issues were corrected, terminology harmonized, limitations expanded, and the Ethical Considerations section updated to clarify GDPR-compliant data handling and storage procedures.

**Reviewer #4**

**Response:**
We thank Reviewer #4 for the positive evaluation and are pleased that the manuscript is now considered acceptable for publication.
